# Design and Efficacy of Nanogels Formulations for Intranasal Administration

**DOI:** 10.3390/molecules23061241

**Published:** 2018-05-23

**Authors:** Blessing A. Aderibigbe, Tobeka Naki

**Affiliations:** Department of Chemistry, University of Fort Hare, Alice Campus, Eastern Cape 5700, South Africa; 200909358@ufh.ac.za

**Keywords:** nanogels, depression, hypertension, influenza, HIV, Alzheimer disease, veterinary vaccine, obesity

## Abstract

Nanogels are drug delivery systems that can bypass the blood-brain barrier and deliver drugs to the desired site when administered intranasally. They have been used as a drug delivery platform for the management of brain diseases such as Alzheimer disease, migraine, schizophrenia and depression. nanogels have also been developed as vaccine carriers for the protection of bacterial infections such as influenza, meningitis, pneumonia and as veterinary vaccine carriers for the protection of animals from encephalomyelitis and mouth to foot disease. It has been developed as vaccine carriers for the prevention of lifestyle disease such as obesity. Intranasal administration of therapeutics using nanogels for the management of brain diseases revealed that the drug transportation was via the olfactory nerve pathway resulting in rapid drug delivery to the brain with excellent neuroprotective effect. The application of nanogels as vaccine carriers also induced significant responses associated with protective immunity against selected bacterial and viral infections. This review provides a detailed information on the enhanced therapeutic effects, mechanisms and biological efficacy of nanogels for intranasal administration.

## 1. Introduction

The treatment of selected diseases such as lifestyle disease, bacterial infections, viral infections and brain diseases is challenging. Lifestyle disease such as obesity is challenging and chronic [1]. It is associated with health risks such as diabetes, cardiovascular diseases, selected cancer and degenerative joint diseases [1]. It is treated by exercise, nutrition, medication and surgery in extreme cases. The drugs used for the treatment act by reducing food intake thereby promoting weight loss. However, some of the drugs suffer from serious side effects such as depression, anxiety and cardiovascular risk [1,2,3]. Obesity has also to be linked to depression, a brain disorder disease [2]. On the other hand, brain diseases are difficult to treat and poses a huge challenge to clinicians. Millions of people are affected by brain diseases worldwide. Globally, diseases of the brain such as Alzheimer’s disease etc. has increased over the past two decades. These diseases result in death and disability. According to Valery et al. the main reasons for the increase in brain diseases are longer life expectancy and the growing population [4]. According to WHO, in 2017, over 47.5 million people were living with dementia, globally. Factors which contributes to brain diseases are environmental, ageing, harmful chemicals, stress, poor nutrition, genetics etc. [5]. Brain diseases are treated by administering therapeutics orally, topically and intravenously. Device-based therapies are also employed such as surgeries, deep brain stimulation and rehabilitation [6]. In some cases, drugs are injected into the brain or via cerebrospinal fluid delivery. However, these techniques are invasive, unsafe, short lasting and local [7,8]. Reconstruction of damaged neural tissue is another approach used to treat brain diseases which is also challenging as a result of neurodegeneration [9]. The blood-brain barrier (BBB) is a barrier that hinders the effective delivery of some therapeutic agents to the central nervous system and inhibits drugs from passing through the endothelial capillaries to the brain [10]. 

Bacterial and viral infections are best managed by vaccination against the infection. However, most of the vaccines require store by refrigeration, are administered using a needle which is not patient compliant, requires multiple administration and is expensive with weak induction of cell-mediated immune response [11]. Developing vaccines for the prevention of viral infections is challenging due to their high rate of viral mutation and antigenic drift [12]. The currently used vaccines efficacy mostly relies on the induction of protective antibodies rather than on cell-mediated immunity [13]. To overcome the limitations associated with the currently used vaccines, nanogels that are potentially safe vaccines carriers with the capability to induce significant antibody responses have been developed [14]. This review will focus on nanogels designed for intranasal administration, their mechanisms and biological efficacy.

## 2. Anatomy of the Nose and Mechanism of Drug Uptake from the Nose-to-the Brain

**Figure 1:**
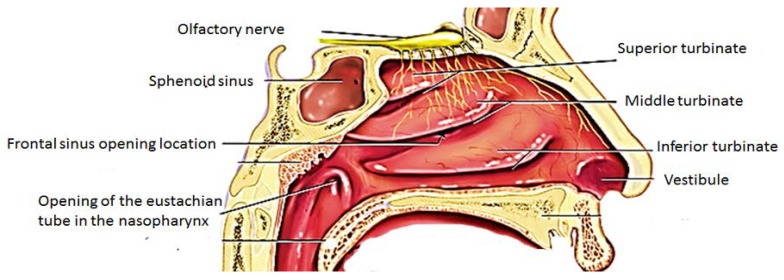
Anatomy of the nose.

The nasal cavity is made up of three regions which are vestibule, olfactory and respiratory regions [15,16] (Figure 1). The nasal vestibule is the anterior region of the nasal cavity surrounded by the cartilages with small hairs known as the vibrissae which filter dust and particle from inspired air and also act as an immunologic response. 

The respiratory region contains the nasal turbinates which project from the lateral wall of each half of the nasal cavity and produce turbulent airflow through the nasal passages ensuring a good contact between the inhaled air and the mucosal surface. The respiratory epithelium is made up of four types of cells which are the basal, goblets, non-ciliated and ciliated columnar cells. These cells are useful in active transport processes, trap moisture keeping the mucosa moist and in mucociliary clearance. The ciliated and non-ciliated cells are covered by non-motile microvilli and are useful for enhancing the surface area and is a region where drug absorption occurs [15,16,17]. The cilia cells are covered with motile cilia and they facilitate mucus transport resulting in mucociliary drug clearance which is predominant in the highly ciliated middle and posterior regions.

The olfactory region is located on the roof of the nasal cavity in humans. The olfactory region is composed of cilia which project down out of the olfactory epithelium into a layer of mucous. This mucous layer is a lipid-rich secretion produced by the Bowman’s glands in the olfactory epithelium [17]. The epithelial layer of the olfactory region is composed of three types of cells: the basal cell, olfactory neural cells and the sustentacular cells. The basal cells originate from the sustentacular cells and they provide mechanical support to other cells. The olfactory neural cells originate at the olfactory bulb and end at the apical surface of the olfactory neuroepithelium and they occupy a space between supporting cells [15,16,17]. Therapeutics with a small size can be transported through axons via the olfactory bulb into the olfactory cortex and then to the caudal pole of the cerebral hemisphere, the cerebrum and the cerebellum. In the olfactory epithelium, the nerve cells project into the olfactory bulb of the brain thereby providing a link between the brain and the external environment and is useful for the transportation of drugs [15,16,17].

The precise mechanisms of drug delivery administered intranasally to the Central Nervous System (CNS) are not fully understood. However, several reports have demonstrated that the nerves connecting the nasal passage to the brain are an important route for drug delivery [16]. Other structures such as the lymphatic system, vasculature and cerebrospinal fluid are also useful in the transport of molecules from the nasal cavity to the CNS [16,17]. The administration of drugs intranasally from the nose to the brain offers several advantages such as enhanced drug absorption through the vascularized mucosa, it bypasses BBB, it is non-invasive, it enhances drug bioavailability, it is patient compliance, and it eliminates gastrointestinal tract and first-pass metabolism and reduces side effects [15].

### 2.1. Mechanism of Drug Delivery from Nose-to-Brain

**Figure 2:**
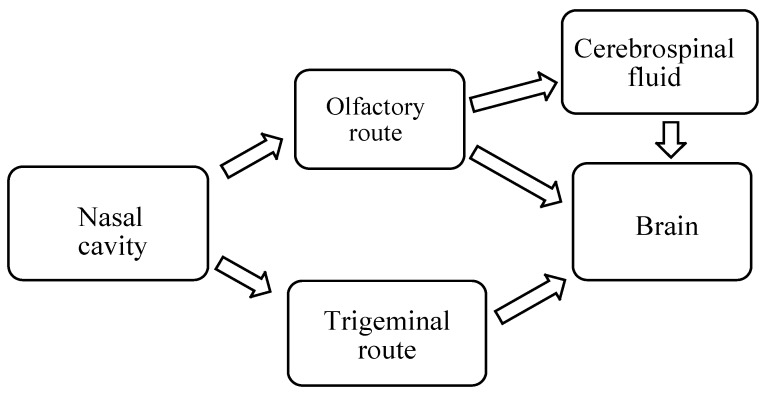
Pathways of nose-to-brain delivery.

There are different pathways via which drugs are transported from the nose to the brain when administered intranasally (Figure 2).

#### 2.1.1. Olfactory Nerve Pathway

**Figure 3:**
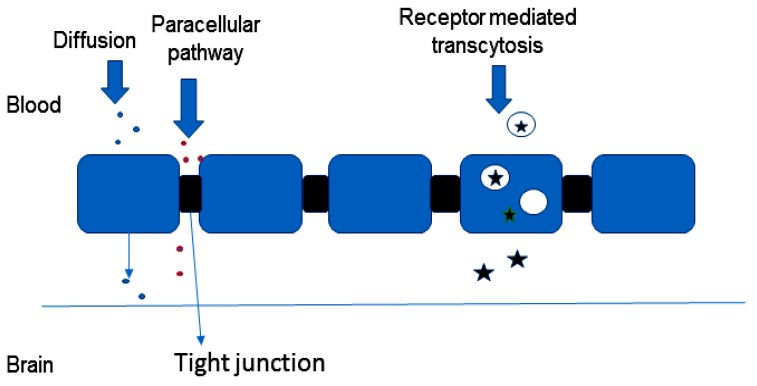
Pathway of therapeutics to the brain.

The different pathways across the olfactory epithelium are transcellular, paracellular and olfactory nerve pathway. In the transcellular pathway, the drug is transported across the sustentacular cells by fluid phase endocytosis, receptor-mediated endocytosis or by passive diffusion (Figure 3). Passive diffusion is rapid and is responsible for the transportation of lipophilic drugs and the rate of transportation is influenced by the nature of lipophilicity of the drug [16,17]. In the paracellular pathway, the transportation of the drug is via tight junctions between sustentacular cells or clefts between sustentacular cells and the olfactory neurons. Hydrophilic drugs are transported by diffusion through aqueous channels. This pathway is slow and the rate of transportation of the drug is influenced by the molecular weight of a drug. In the olfactory nerve pathway, the drug is taken up by endocytosis or pinocytosis into the neuronal cell and transported to the olfactory bulb by the intracellular axonal transport [16,17,18].

#### 2.1.2. Trigeminal Nerve Pathway

The trigeminal nerve is the largest cranial nerve and it supplies nerves to the respiratory and olfactory epithelium of nasal passages and enters the CNS. A portion of trigeminal nerve ends in the olfactory bulbs. The trigeminal nerve is useful for the communication of sensory information. Ophthalmic and maxillary branches of trigeminal nerve play an important role in the nose to brain drug delivery because the neurons from the ophthalmic and maxillary branches pass directly through the nasal mucosa [16,19]. Trigeminal nerve enters the brain from the respiratory epithelium of the nasal passages through anterior lacerated foramen and the cribriform plate near the olfactory bulb thereby producing entrance into both rostral and caudal region of the brain. However, it is important to mention that the facial nerve and the Grueneberg ganglion may also provide a route via which drugs administered intranasally reaches the CNS [16,19].

#### 2.1.3. Other Pathways

Other pathways involved in the transportation of drug from the nose-to-the brain is the cerebrospinal fluid and nasal lymphatics. In the aforementioned pathways, drugs are transported from the nasal passage to the cerebrospinal fluid to the brain with no significance entry into the blood [16]. This route of drug transportation is influenced by factors such as the degree of ionization of drugs, lipophilicity and molecular weight [16,19]. 

## 3. Nanogels Designed for Drug Delivery to the Brain

Nanogels are hydrogel materials with a three-dimensional network in the nanoscale size [20]. They are crosslinked, swellable networks that can hold a large capacity of water [20]. They can be prepared from biopolymers, synthetic polymers or the combination of both. Their physicochemical properties can be tailored by modifying their chemical composition such as degradability, charge, softness, size, porosity and amphiphilicity. They occur in different shapes and are composed of a core-shell or a core-shell-corona structure with a crosslinked layer useful for structural integrity [20]. They are hydrophilic, biocompatible, have a high drug loading capacity, useful for targeted delivery, exhibit stimuli-responsive behaviour which protects the loading bioactive agent from degradation making them suitable for biomedical applications such as drug delivery, in diagnostic tool etc. [21,22,23,24]. 

In the design of nanogels, the precursors used influenced their properties. In order to modify the degradability of nanogels, labile bonds are integrated into the polymer backbone or in the crosslinks of the hydrogel network resulting in nanogels which are both reductive and also acid-sensitive and degrade under these physiological conditions [24,25]. Their high stability is attributed to internal crosslinking [25]. In their swollen state, a large inner space in the network is present for drug loading resulting in high drug loading capacities [24]. Their swelling ability is influenced by factors such as polymer matrix chemical structure, the degree of crosslinking, external triggers and charge density. When they swell, solvent penetrates into the free spaces resulting in a change in the volume which may be rapid or slow depending on the degree of crosslinking [24]. Their capacity to swell and collapse is useful for optimal drug loading and release [24]. Their soft nature is unique and influences their bio-distribution resulting in longer circulation in the body [24,26,27,28]. They can also pass through physiological barriers [24,26,27,28,29,30]. Their small size makes them able to target the desired sites resulting in enhanced cellular uptake [24,26,27,28,31,32]. Drug release from nanogel is influenced by factors such as degree of crosslinking of the gel, the degradation rate of the network, the interaction of the drug with the polymer chains, the molecular weight of the polymer and the gel network degradation rate [24].

## 4. Preparation of Nanogels

The preparation approach of nanogels can be classified as physical or chemical crosslinking [20,24]. In physical crosslinking, interactions such as van der Waals, hydrogen bonds, an electrostatic bond which involves entanglements of polymer chains occur [20,24]. Chemical crosslinking involves crosslinking by covalent bonds resulting in a stable and rigid polymer network [20,24]. The stability of the network is influenced by the nature of the polymer matrix, the chemical composition of the matrix, crosslinking type of the polymer chains and the particle size. However, it is important to mention that chemically crosslinked nanogels are more attractive in biomedical applications due to their size stability and reproducibility when compared to the physically crosslinked nanogels that are not stable and their size cannot be controlled during synthesis. Physically crosslinked nanogels involves modifying the concentration of the polymer and other features such as pH, temperature and ionic strength [24,33]. Physically crosslinked nanogels are formed under mild conditions and they are fragile [33]. Chemically crosslinked nanogels are prepared by polymerization reactions such as heterogeneous polymerization involving the use of crosslinkers, conventional and radical polymerization. Initiators are employed in order to incorporate functionalities useful for bio-conjugation [20]. Other crosslinking methods used for the chemically crosslinked nanogels are Schiff-base, click chemistry, amide crosslinking, enzyme-based crosslinking, etc. [20,34,35]. 

### 4.1. Nanogels Application for Intranasal Administration

Nanogels have been developed for the intranasal administration of drugs for the treatment of lifestyle disease, as vaccine carriers and for the treatment of brain diseases such as Alzheimer disease, depression and migraine etc. Nanogels can be used to deliver hydrophilic and hydrophobic drugs and the intranasal administration of the drugs can bypass BBB making them useful for the treatment of brain diseases. The application of nanogels for drug delivery have been found to be more effective when compared to the administration of free drug such as reduced dug toxicity, enhanced cellular uptake of the drug, high drug loading and the release of the loaded drug in a controlled manner at the targeted site [24,25,29,30,31]. However, the type of material used to prepare nanogels for intranasal administration can result in loss of epithelial cell, the shrinkage of the mucosal layer and loss of the ciliary layer [36,37].

#### 4.1.1. Nanogels Application for Intranasal Administration of Anti-Alzheimer Drugs

Alzheimer’s disease (AD) is a brain disease that is characterized by the presence of extracellular senile plaques composed primarily of amyloid β peptide and intracellular neurofibrillary tangles formed by Tau protein [38]. The neurofibrillary tangles are composed of hyperphosphorylated Tau protein which is found in the axons of neurons which is attached to the microtubules. In a hyper-phosphorylated state, Tau does not bind to the microtubules thereby resulting in cell collapse [39]. Recently, insulin resistance and insulin action have been linked to AD and this condition is referred to as Type 3 Diabetes [40,41]. Insulin and insulin receptors in the brain in the hippocampus and cerebral cortex is useful in learning and memory. Reports further revealed that the reduced levels of insulin receptors in postmortem cerebral material of AD patients confirmed that insulin signalling is linked to cognitive and functional brain deficits in the elderly [42,43,44]. Obesity, Type 2 diabetes and other metabolic pathologies are risk factor for AD [45,46,47]. Alzheimer’s disease is linked with insulin resistance of the brain, that is the reason it is now referred to as type 3 diabetes [45,46,47].

Picone et al. prepared poly(*N*-vinyl pyrrolidone)-based nanogels prepared by e-beam irradiation with a covalent attachment of insulin for brain delivery [48]. Intranasal administration of the formulation revealed the absence of immunogenic response of the nasal mucosa in vivo which was evident by the levels of IL-6, IL-1β, IL-10 and TGFβ in mouse total brains which were comparable with the controls. The brain uptake of the formulation was enhanced when compared to the administration of the free insulin. A significant amount of the insulin was found in the anterior and cerebellar regions of the mouse brain suggesting that the olfactory and trigeminal nerve pathways are involved in the transportation of the drug to the brain [48]. In another report by Picone et al. carboxyl-functionalized poly(*N*-vinyl pyrrolidone)-based nanogel system was prepared by ionizing radiation with covalent attachment of insulin [44]. The nanogel protected the incorporated insulin from protease degradation resulting from the ability of the flexible polymeric structure of nanogel to conform differently in different environments. In aqueous solution, nanogel produce transient hydrophobic pockets thereby protecting the incorporated drug. The binding capability of the formulation to insulin receptor resulted in a trigger of insulin signalling via AKT activation. Insulin conjugated to the nanogel activated AKT an hour after addition to cell culture reaching a maximum effect after 3 h and the activation level obtained was twice of the free insulin. Furthermore, in vitro evaluation revealed that the formulation crossed the BBB when compared to free insulin indicating that the drug utilized the paracellular pathway. The finding suggests that intranasal administration of insulin using nanogels can transport the drug from the olfactory mucosa to the olfactory bulb via the olfactory nerve pathway resulting in rapid delivery to the CNS. The formulation was able to exhibit neuroprotective effect against the toxicity induced by the amyloid peptide in vitro [44]. Both reports revealed that nanogels are potential delivery systems that be used for the management of Alzheimer disease because insulin administration to AD patients (mild or moderate) improves their cognitive performances, brain function and preserve the rate of glucose utilization of the brain (Table 1) [49]. Furthermore, the ability of the formulation to activate insulin signalling also provides a neuroprotective mechanism that counters inflammation, oxidative stress, mitochondrial damage and neurodegeneration [50]. The irradiation method used for the preparation of the nanogels enhance intramolecular, intermolecular crosslinking, grafting of the polymer and the sterilization of the nanogels [51]. The capacity of the nanogel to cross the nasal epithelium is attributed to factors such as the protease resistance, mucoadhesive properties, and their ability to weaken the tight junctions [44]. Elnagger et al. incorporated piperine, a phytopharmaceutical useful for neuroprotective in Alzheimer’s disease onto hydrogel-based nanoparticles [52]. Piperine is hydrophobic and is limited by a pre-systemic metabolism. The % drug entrapment efficiency of the formulation was 81.70% with a particle size of 249 nm and zeta potentials of +56.30 mV. In vivo studies showed that the formulation enhanced the cognitive functions of the mice which were comparable to donpezil injection, a medication used for the treatment of dementia in Alzheimer patients. Piperine is limited pharmacologically by symptoms such as nasal irritation and brain toxicity that exhibits in some of the patients [52]. Its hydrophobic nature results in drug clogging in the olfactory bulb and a poor rate of delivery. Loading the drug onto the nanoparticle was able to overcome the clogging of the drugs in the olfactory bulb. The mode of delivery of the drug from the hydrogel nanoparticles is believed to be via the olfactory pathway with permeation and diffusion of the drug into the cerebrospinal fluid thereby enhancing the cognitive effects [52].

#### 4.1.2. Nanogels Application for Intranasal Administration of Anti-Schizophrenia Drug

Schizophrenia is a chronic mental health disorder which is characterized by hallucinations, disorganized behaviour etc. [53,54]. It is caused by deficiency or excess of neurotransmitters such as dopamine, glutamate and serotonin. Factors which are genetic and environmental also risk factors for the disease. The condition is managed using antipsychotic drugs which suffer from adverse effects such as diabetes mellitus, weight gain, hyperlipidemia which increase the risk of cardiovascular mortality, dystonia, cataracts, sexual dysfunction etc. [53]. Due to the disorganized behaviour associated with the disease, many of the patients are in denial of the illness resulting in non-compliance with medication. Olanzapine is an antipsychotic drug used for the treatment of schizophrenia and it is administered orally. However, the drug exhibit low bioavailability due to the first-pass metabolism in which over 40% of the drug is metabolized before it reaches the systemic circulation. In order to overcome its bioavailability limitation, Baltzley et al. prepared hydrogel nanoparticles by ionotropic gelation process of chitosan with pentasodium tripolyphosphate anions loaded with olanzapine (Table 1) [55]. The percentage drug loading was 17% with an encapsulation efficiency of over 90%. Average particle size was in a range of 179–237 nm. In vivo studies indicated that administering the nanoparticles intranasally significantly enhanced the drug systemic absorption with an absolute bioavailability of 51% when compared to intravenous administration of the drug which was 28% revealing that the nanoparticles enhanced the nasal absorption of the drug. Chitosan dissolution rate in release medium, its ability to adhere to mucosal tissues and its capacity to transiently open the tight junctions influenced the drug absorption of the drug from the nanoparticles on the administration of the formulation intranasally [55,56]. The finding revealed that nanogels are potential devices for administration of antipsychotic drugs to non-compliant patients that avoid injections.

#### 4.1.3. Nanogels Application for Intranasal Administration of Anti-Migraine

Migraine is a common brain disorder. There are currently three treatment approach which are lifestyle and trigger management, acute treatments to manage attacks and preventive treatment to reduce the tendency of having attacks [57]. Hu et al. loaded lidocaine hydrochloride, a commonly used drug for the treatment of migraine onto nanogels for intranasal administration (Table 1) [58]. However, lidocaine hydrochloride is limited pharmacological due to its rapid nasal mucociliary clearance which impede its efficacy. The nasal gel was prepared using hydroxypropyl methyl cellulose. In vivo studies in rat nasal mucosa model showed that the drug-loaded nasal gel was not toxic to the mucocilia. The absolute bioavailability of the formulation was 1.5 times compared to the nasal spray. The drug targeting index of olfactory and ventricle after nasal gel was 2.15 and 1.51 compared to spray administration that was 1.66 and 1.26, respectively. The finding suggested that the drug was transported from the nasal cavity to the central nervous system [58]. The enhanced bioavailability of drug loaded onto nanogel indicate that the nanogel reduced the rate of nasal mucociliary clearance which is a unique feature of the nanogel for enhanced efficacy of the drug. The nanogel also enhanced the targeting delivery of the loaded drug which was confirmed by the high uptake of the drug in the brain of the mice in vivo.

#### 4.1.4. Nanogels for Intranasal Delivery of Anti-Depression Drug

Depression is a brain disorders caused by the disturbances in the balance of norepinephrine, serotonin and dopamine level [59,60]. The treatment of depression require a continued presence of the anti-depression drug in the brain over an extended period of time in order to maintain a steady level at the site of action [59]. Venlafaxine is an antidepressant drug which increases the diminished level of neurotransmitters in the synaptic cleft and is effective than selective serotonin reuptake inhibitors in the treatment of depression and anxiety disorders [59]. It has an elimination half-life of 4–5 h thereby requiring frequent administration in order to maintain its therapeutic concentration. In the liver, it undergoes first-pass metabolism resulting in a slow onset of action [59]. It is hydrophilic in nature and limits its permeability and poor antidepressant action in the brain revealing the need to overcome the limitation of poor permeation at the site of action [59].

Dange et al. prepared nanostructured lipid-based gel for the intranasal drug delivery of venlafaxine by emulsion solvent diffusion and evaporation method (Table 1) [61]. Properties of nanostructured lipid carriers such as particle size and its distribution, % Drug loading were investigated. The carrier dispersion was suitably gelled and characterized for drug content, pH, viscosity, in-vitro and ex-vivo release and in-vivo pharmacodynamic study. FTIR and DSC analyses confirmed the absence of any significant chemical interaction between Venlafaxine and excipients in the formulation. The particle size of was 51.83 nm with a zeta potential of − 8.08 mV. The gel exhibited a rapid onset with a prolonged duration of action when compared to the pure drug solution [61]. The prolonged duration of action of the formulation indicates that nanogels are patient compliance and will not require continuous taking of the drug orally. Oral administration of the formulation hinders the first-pass metabolism in the liver that results in a slow onset of action of venlafaxine which is also overcome when loaded onto nanogels resulting in a rapid onset of action of the drug. Hague et al. prepared alginate nanogels loaded with venlafaxine for intranasal administration [62]. The particle size was 173.7 nm with a positive zeta potential of +37.4 mV. The positive zeta potential facilitates the adhesion and transport properties of the nanogels resulting from the electrostatic attractions between the positively charged formulation and the negatively charged cell membranes. The in vitro release of drug from the nanogel was 86% over a period of 24 h indicating a controlled drug release mechanism. Ex vivo permeation studies on isolated porcine nasal mucosa showed increased permeation of drug and the mucosal absorption is attributed to an interaction of a positively charged amino group on the alginate with negatively charged sialic acid residues on the cell membranes and tight junctions of the mucosal epithelial cells allowing the opening of the tight junctions [63]. In vivo studies in Wistar rats revealed that the concentration of the drug in the plasma was lower in the rat administered with the formulation intranasally when compared to the rat administered intravenously. The drug concentration in the brain was high in the brain administered with the formulation intranasally with a brain/blood ratio of 0.1091 compared to the free drug administered intravenously and intranasally which was 0.0293 and 0.0700, respectively. The high brain/blood ration of the drug after administration of the formulation intranasally revealed the direct nose to brain transport of drug bypassing the BBB. The brain concentration of the drug after administration intranasally was (743 ng/mL; *t*_max_ 60 min) which was significantly higher than the drug solution administered intravenously (383 ng/mL; *t*_max_ 30 min) and drug administered intranasally (397 ng/mL; *t*_max_ 60 min). The olfactory and trigeminal nerve systems were believed to be involved in the nose to brain transportation of the drugs to the olfactory bulb or trigeminal region in which the drug enter into other brain regions by diffusion thereby bypassing systematic circulation [62]. Singh et al. prepared thiolated chitosan hydrogel nanoparticles for the nasal delivery of selegiline hydrochloride by ionic gelation method. The particle size, entrapment efficiency and zeta potential of nanoparticles was found to be 215 nm, 70%, and +17.06 mV, respectively. The formulation significantly attenuated oxidative stress and restored the activity of the mitochondrial complex in vivo revealing its potential as a carrier for delivery of anti-depressant drug [64].

### 4.2. Nanogels Application for Intranasal Delivery of Anti-Hypertensive Drug

Amlodipine is a calcium channel blocker that is used to improve vascular selectivity [65]. It binds to the target receptors resulting in a smooth onset of action with a 24 h control of blood pressure. It is commonly used in the treatment of heart diseases such as angina and hypertension. It acts by relaxing the vessels thereby resulting in the easy flow of blood. It is administered orally and this result in abdominal pains in some patients. Kamble et al. reported nanostructured lipid gel for the intranasal delivery of amlodipine besylate, an antihypertensive drug [66]. The gel was prepared by an evaporation method and emulsion solvent diffusion (Table 1). The particle size, zeta potential and entrapment efficiency of the formulation was 424.3 nm, −14.2 mV and 53%, respectively. The drug content loaded onto the carriers gel was 97%. The formulation did not exhibit drug toxicity on the sheep nasal mucosa in vitro revealing its potential application for intranasal administration [66]. The high drug entrapment of the nanogel indicate its potential for targeted drug delivery, extended drug release and improve drug bioavailability. Its non-toxic nature on the nasal mucosa further revealed that the nanogel formulation administered intranasally can overcome the abdominal pain that is a common side effect when amlodipine besylate is administered orally.

### 4.3. Nanogels Application for Intranasal Administration of Anticancer Drugs

Leuprolide acetate is a therapeutic peptide that is used to treat cancer such as prostate cancer and breast cancer [67]. Leuprolide acetate is available as depot formulations for subcutaneous administration every month or three months requiring daily injections [68]. However, it exhibits low drug bioavailability when administered to the nasal cavity resulting from low membrane permeability, mucociliary clearance due to short residence time and high enzymatic reaction in the epithelium [67]. Shanaz et al. investigated the application of thiolated-chitosan hydrogel nanoparticles for the delivery of leuprolide acetate [67]. The prepared nanoparticles loaded with leuprolide acetate zeta potential was +10.9 mV signifying its ability to exhibit a strong electrostatic interaction with negatively charged mucus layer. The payload of the drug onto the nanoparticles was 12%. A sustained drug release profile of 43% was released from the nanoparticles over a period of 2 h suggesting that the release of the drug is slow during the residence in the nasal cavity. The beat frequency evaluation revealed the absence of toxicity of the formulation. Incorporating the drug onto the nanoparticles resulted in a 6.9-fold increase in area under the curve, 4.5-fold increase in mean residence time, more than 4-fold increase in elimination half-life, 3.8-fold increase in maximum plasma concentration, and a 7-fold decrease in plasma clearance rate compared to the nasal solution alone. The mode of delivery of leuprolide acetate from the nanoparticles across the nasal epithelium was via paracellular pathway [67]. Thiolated polymers have been reported to offer several advantages as drug delivery systems for intranasal administration such as extended residence time, enhanced permeation effect resulting in the opening of tight junctions, inhibition of protein tyrosine phosphatase and they are non-toxic [69,70,71].

### 4.4. Nanogel Application for Intranasal Delivery of Anti-HIV Drug

HIV is a viral infection and the currently approved drugs for the treatment of the infection do not attain adequate drug concentrations in the central nervous system resulting in their inability to suppress viral replication in the brain completely [72]. Reports have shown that the CNS act as a reservoir for the virus after systemic eradication [73,74,75]. Didanosine is an HIV reverse transcriptase inhibitor that is administered orally. It exhibit low bioavailability after oral administration with limited CNS penetration resulting from its acid lability and polar nature, first-pass liver metabolism and low intestinal permeability [76]. Al-Ghananeem et al. developed chitosan-based hydrogel for intranasal administration of didanosine in order to enhance the bioavailability of the drug (Table 1) [76]. The average particle size of the nanoparticles were in the range of 269–382 nm and the drug loading capacity ranged from 9% to 47% with an encapsulation efficiency of 95%. The plasma concentration of the drug after intravenous administration of the formulation was 4591 ng/mL which declined rapidly over a period of 30 min. After intranasal administration of the formulation, the drug plasma concentration in the first 15 min was low compared to the intravenous administration [76]. The drug concentration in the cerebrospinal fluid, olfactory bulb and brain in vivo was high after administration of the formulation intranasally when compared to intravenous administration. The high uptake of the drug in the brain after intranasal administration is attributed to chitosan nanoparticles ability to adhere to the mucosal tissues thereby opening the tight junctions resulting in paracellular transport. The small diameter of the nanoparticles resulted in the transportation of the drug by transcellular pathway via olfactory neurones to the brain via the various endocytic pathways of sustentacular or neuronal cells in the olfactory membrane. The ability of the anti-HIV agents to reach the CNS from the nanoparticles suggest that the formulation is a potential therapeutic which can overcome progressive deterioration in mental function accompanying AIDS and can also suppress significantly viral replication in the brain.

### 4.5. Nanogels Application as Vaccine Delivery System for Intranasal Administration

**Figure 4:**
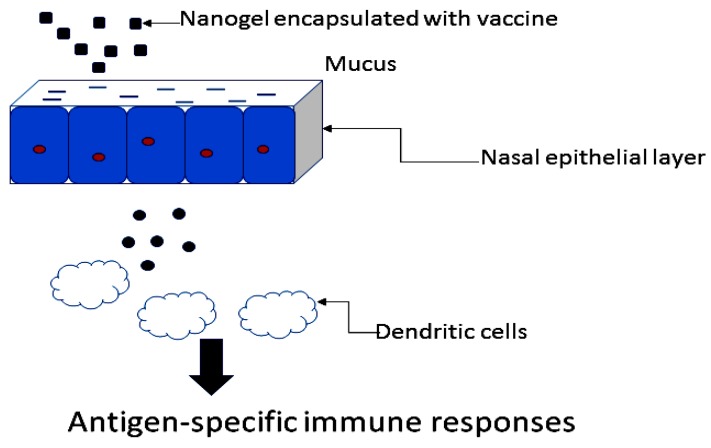
Nanogel as vaccine carriers.

Nanogels properties such as size, surface charge, shape, molecular weight of polymers used, hydrophilic and hydrophobic property are factors that can induce immunity. In the application of nanogels as vaccine delivery systems, the particle sizes of the nanogel influences the drug transport across the mucosal surfaces. For effective antigen delivery, the nanogel must gain access to the mucosal epithelia (Figure 4). Delivery systems of particle size of 50 nm can diffuse in mucus freely [17]. The size of the system influences their immunological activity. Surface charge of carriers influences properties such as the bioadhesiveness, entrapment efficiency, stability and in vivo immunogenic performance of a vaccine formulation [17,77]. Nanogels have been used as vaccine delivery systems for the prevention of hypertension, obesity, bacterial and viral infections.

#### 4.5.1. Nanogels as Vaccine Delivery System for Intranasal Administration for the Prevention of Bacterial Respiratory Infection Caused by *Streptococcus pneumoniae*

The brain is protected from bacterial invasion by features such as the skull, the pia, the dura, the arachnoid membrane and the glia limitans [78,79]. However, bacterial infections can spread via the bloodstream to the brain. Bacteria can penetrate into the brain from infected adjacent air sinuses, the middle ear and the mastoids [78,79]. Bacterial infection such as meningitis is caused by *Streptococcus pneumoniae* and *Neisseria meningitidis*. Bacterial meningitis occur after bacterial colonization of the nasopharyngeal or middle ear after which the bacteria get into the blood. The bacteria invade the tissue and intravascular space. The cerebrospinal fluid is a media in which the bacteria multiply. The bacterial toxins cause neuronal apoptosis, and endotoxin released from the bacteria enhance clotting resulting in disseminated intravascular coagulation. Cells of the innate immune system of the brain which are found in the BBB etc. release cytokines, chemokines, and complement when it detect bacteria which attract granulocytes into the cerebrospinal fluid to kill bacteria [79]. However, these granulocytes have a short life span. Brain damage in bacterial meningitis is caused by the direct action of bacteria and antibacterial inflammatory response [79]. The attachment of the bacteria to brain endothelium is the initial step in the bacteria penetration of the BBB thereby disrupting the endothelium junction complexes and compromising the integrity of BBB making the infection difficult to be treated [80].

Pneumococcal-based respiratory infection is caused by the bacterium *Streptococcus pneumoniae* [81]. It is found in the upper respiratory tract of healthy people. It is managed by vaccination and antimicrobial therapy. The bacteria that causes the disease colonizes the nasopharynx and can spread via the airway to the lower respiratory tract thereby causing pneumonia. The bacteria can also penetrate the epithelial cell surface resulting in local infection [81]. The bacteria demonstrate transformation resulting in a high rate of variation in the genome. It acquires environmental DNA from many sources including the extracellular matrix of pneumococcal biofilms and microbial fratricide in which specifically siblings are lysed, enhancing the release of chromosomal DNA [81]. The recommended first-line antibiotics used for the treatment of the infection is amoxicillin and cephalosporins. The risk factors for the infection in children are poor nutrition, lack of breastfeeding, exposure to air pollution, HIV infection, premature birth, overcrowding and poor condition [82]. HIV-infection is an important risk factor in children in sub-Saharan Africa where the burden of paediatric HIV disease is concentrated [82]. However, the factor that makes the treatment not to be effective is drug resistance.

Kong et al. developed a pneumococcal vaccine from pneumococcal surface protein A using a nanogel composed of a cationic cholesteryl group-bearing pullulan for intranasal administration and for the prevention of *S. pneumoniae* infection (Table 1) [83]. Most subunit type vaccines are poor immunogens for the induction of antigen-specific immune response in both systemic and mucosal immune compartments when administered nasally. The aforementioned limitation can be overcome by employing nasal delivery systems such as nanogel. In vivo studies by intranasal vaccination of the formulation on female BALB/c mice once weekly over a period of three weeks was studied. The mice vaccinated with the formulation survived the lethal challenge with *S. pneumoniae* when compared to the mice vaccinated with nanogel complexed a recombinant nontoxic receptor-binding fragment of *Clostridium botulinum* type A neurotoxin subunit antigen. The respiratory tracts of mice immunized with the formulation displayed less colonization and invasion of pneumococcal organisms. The formulation enhanced responses associated with protective immunity against pneumococci such as Th17, mucosal IgA and systemic IgG antibody responses. The mode of action of the nanogel formulation is believed to be attributed to the serum and BALF IgGs antibody which was induced by the formulation in the lower respiratory compartment resulting in the mice survival against the lethal challenge with *S. pneumoniae*. The serum antigen-specific IgG antibodies are useful for the prevention of invasive disease associated with clinical signs while antigen-specific sIgA antibodies are important for the prevention of colonization of the upper respiratory tract by *S. pneumoniae*. The formulation induced Th17 cells, a hallmark of autoimmunity in the nasal passage, systematic region and draining lymph nodes confirming the formulation induced humoral and cellular immune responses, indicating its potential application as a carrier for mucosal vaccine [83]. The nanogel without the vaccine did not activate immune cells because of the absence of biologically active adjuvant and it further indicated that the nanogel acted as a carrier in which the antigens released from the nanogel is taken up in the dendritic cells nasal mucosa resulting in antigen-specific immune responses. Fukuyama et al. prepared self-assembled nanosized nanogel composed of a cationic type of cholesteryl group-bearing pullulan for efficient delivery of an antigen to the epithelial cells in the nasal cavity [84]. The formulation was [^18^F]-labelled vaccine formulation of the nanogel and it was retained for a period of 6 h in the nasal cavity and was not transported to the brain after nasal administration in non-human primates, the macaques confirming its safety. Recently, there has been concern that vaccine administered intranasally can deposit in the CNS via transportation of the vaccine from the nasal cavity to the brain via the olfactory pathway. These results indicated that the nanogel-vaccine formulation was taken up into the nasal epithelium by endocytosis followed by the release of antigen from the nanogel in the epithelium followed by drug release from the nasal epithelium by exocytosis in which the drug is taken up by the nasal dendritic cells. The formulation induced mucosal antigen-specific mucosal IgA and systemic IgG Ab responses in the macaques which are important in the protection against respiratory pathogens such as *S. pneumoniae* and survival against lethal challenge with *S. pneumoniae*, respectively. The formulation induced humoral and cellular immune responses in macaques which were evident by enhanced expression levels of miR-181a and miR-326, candidate miRNA biomarkers for induction of mucosal immunity [84].

Yuki et al. prepared cationic-based cholesteryl group-bearing pullulan nanogel loaded with Pneumococcal surface protein A antigen as a pneumococcal intranasal vaccine [85]. The vaccine induced nasal and bronchial secretory IgA (SIgA) antibodies and antigen-specific serum IgG, significantly. The levels of Pneumococcal surface protein A antigen-specific antibodies were comparable with mice nasally immunized with free Pneumococcal surface protein A antigen and cholera toxin, a mucosal adjuvant. The nanogel-vaccine formulation also induced Pneumococcal surface protein A antigen-specific immune responses that provide protection against the lethal challenge with *Streptococcus pneumoniae* Xen10 in vivo. The mice that were administered the nanogel-vaccine formulation exhibited fewer numbers of pneumococcus on the surface of the bronchial mucosa signifying protection from the pneumococcal invasion of the lung parenchyma. These results revealed the efficacy of nanogel-based as a nasal vaccine system against the respiratory infection of pneumococcus [85].

#### 4.5.2. Nanogels Application as Vaccine Carrier for Intranasal Administration for the Prevention of Viral Respiratory Disease

Influenza is an acute viral respiratory disease. Two classes of antiviral drugs that are used to treat influenza are adamantine derivatives which include amantadine and rimantadine and the neuraminidase inhibitors such as zanamivir and oseltamivir. These drugs act by inhibiting the activity of the protein in the influenza virus. However, these drugs suffer from drug resistance to seasonal influenza virus [86,87]. Nochi et al. developed an intranasal vaccine-delivery system nanogel formulation composed of a cationic type of cholesteryl-group-bearing pullulan loaded with a non-toxic subunit fragment of *Clostridium botulinum* type-A neurotoxin for intranasal administration (Table 1) [88]. Intranasal immunization with the vaccine effectively induced *Clostridium botulinum* type-A neurotoxin-specific mucosal IgA antibody responses which were significant in the lamina propria and paranasal sinuses of the nasal passages when compared to the naked *Clostridium botulinum* type-A neurotoxin. The vaccine did not accumulate in the brain revealing its safety. The nanogel conveyed the vaccine antigen into the respiratory immune system and it did not provide an adjuvant-like activity to dendritic cells. In vivo tracer study with [^111^In]-labelled formulation of the vaccine administered intranasally revealed that the vaccine-formulation was not transported to the olfactory bulbs or brain revealing its safety [88]. Mice immunized intranasally with the nanogel-vaccine formulation survived without any severe side effects. However, the mice immunized with the free vaccine developed neurological signs resulting in the death of the mice. The nanogel-vaccine formulation was internalized into the nasal epithelium immediately after the intranasal administration followed by the release of the loaded vaccine in a controlled release profile in the nasal epithelial cells. The nanogel acts as a carrier to convey the vaccine antigen into the respiratory immune system effectively leading to the induction of antigen-specific respiratory immune responses. The nanogel does not provide an adjuvant-like activity to dendritic cells which was visible by the non-expression of co-stimulatory and antigen-presentation molecules. Nagatomo et al. incorporated tumor necrosis factor-α, a proinflammatory cytokine, into cholesteryl pullulan-based hydrogel nanoparticles as a vaccine adjuvant for intranasal administration [89]. The formulation induced systemic IgG1 and mucosal IgA antibodies. Combination of the conventional split vaccine with the formulation was effective in protecting the mice against a lethal challenge of A/PR/8/34 (H1N1) influenza virus suggesting that the formulation induced broad cross-protection. The formulation did not result in toxic side effects [89].

### 4.6. Nanogels Application as Vaccine Delivery System for Intranasal Administration of Vaccine for the Prevention of Obesity

Obesity is a serious health challenge and it is associated with multiple complications such as cardiovascular diseases, diabetes, osteoarthritis, sleep apnea, some cancers such as breast, prostate and colon cancer [90]. However, the treatment approach of obesity is insufficient in terms of efficacy. Weight loss can lead to physiological adaptations that result in weight gain [91]. Weight loss by lifestyle treatment is difficult to sustain. Several anti-obesity drugs have been approved such as GABA_A_ receptor activators, sympathomimetics, glucagon-like peptide-1 (GLP-1) receptor agonists, pancreatic lipase inhibitors, an opioid antagonist, dopamine-norepinephrine reuptake inhibitor etc. [91]. However, these drugs suffer from side effects resulting in greater weight loss. Many patients do not use these drugs because of the concerns about safety and efficacy [91]. Treating of obesity in the elderly is even more challenging. Inducing weight loss in elderly patients improves their quality of life and reduces medical complications. However, choosing treatments that reduce weight with simultaneous minimal loss of muscle tissue and bone tissue can result in an increased risk of fractures [90,92].

Azegami et al. developed a vaccine for the prevention of obesity containing ghrelin as the vaccine antigen and cyclic di-GMP as the adjuvant for intranasal administration (Table 1). Ghrelin is an endogenous peptide composed of a ligand for growth hormone secretagogue receptor and amino acids [93]. *N*-octanoylation at serine 3 residue, is essential for ghrelin to activate growth hormone secretagogue receptor. Systemic immunization with these vaccines in vivo in pig and rat resulted in weight gain [93,94,95]. Octanoyl modification of serine 3 residue of ghrelin is unstable resulting in its rapid hydrolysis in the body suggesting that a vaccine that can be administered non-invasively without the acylation at serine 3 residue is a potential carrier for the vaccine of ghrelin [93]. In vivo studies of the vaccine revealed that despite the lack of acylation at serine 3 residue of the ghrelin sequence in ghrelin-pneumococcal surface protein A antigen, intranasal immunization of the vaccine-induced des-acyl ghrelin specific antibodies and ghrelin-specific serum IgG antibodies. The main targets of the induced serum IgG antibodies were the N-terminal (amino acids 1–9) and the C-terminal (amino acids 20–28) portions of the ghrelin sequence. The vaccine was administered intranasally to diet-induced and leptin-deficient mice once a week over a period of 5 weeks. One or two doses of vaccine did not induce ghrelin-specific antibody responses. However, serum IgG antibody against ghrelin was elicited when intranasal immunization was given three or four times. Five doses of immunization induced a high anti-ghrelin antibody titer. Administering three doses of nasal vaccine did not affect the body weight gain in diet-induced obese mice suggesting that five doses of intranasal immunization were the most suitable dose for reducing the effect of obesity.

Five doses of the vaccine maintained a high titer for antibodies against ghrelin for 1 year. However, the reduction effect on the body weight gain was lost at the 9 week after the final vaccination in diet-induced obese mice. This finding suggest that the antibody titer at 9 weeks after the final immunization was insufficient when compared to the titers in the previous weeks and an accelerated weight gain in diet-induced obese mice after 9 weeks occurred due to high-fat diet. Ghrelin-specific antibody is insufficient to reduce the body weight gain and studies have proven that long-term consumption of a diet high in fat in mice can cause refractory obesity that is not fully reversible when the amount of fat in the diet is reduced [96]. Intranasal immunization of the vaccine reduced the body weight gain in diet-induced mice by 9.5% compared to the control diet-induced mice at 4 weeks of being fed a high-fat diet. The vaccine also decreased body weight in a leptin-deficient genetically obese mouse. The vaccine did not alter food intake in immunized mice suggesting that the vaccine may be more effective when used in combination with dietary restriction. Peroxisome proliferator-activated receptor gamma expression in adipocytes was increased in the mice immunized with the formulation which further revealed that the vaccine can also prevent adipocyte hypertrophy and insulin resistance induced by a high-fat diet [93].

### 4.7. Nanogels as Vaccine Delivery System for Veterinary Applications

Nanogels have been reported to be employed as vaccine delivery system for the prevention of parasitic and viral infectious diseases in animals.

#### 4.7.1. Nanogels Application as Vaccine for Intranasal Administration for the Prevention of Encephalomyelitis

Encephalomyelitis in dogs is caused by Neospora caninum, an intracellular parasite. The parasite is also reported in various species of livestock including cattle, sheep, goats, horses and deer [97,98]. The parasite is not known to infect humans. The parasite is a significant veterinary public health problem and causes abortion, reduced milk yield, premature culling and reduced post-weaning weight gain in beef calves. The parasite can be transmitted to cattle after ingestion of oocysts via contaminated feed or water. It can also be transmitted via the placenta from mother to foetus [97]. Debache et al. prepared nanogel and the encapsulation of recombinant NcPDI. In vivo studies was performed by the vaccination of the mice intranasally or intraperitoneally routes. Nanogels were prepared from chitosan and alginate. The mice that were administered with the formulation intranasally survived from the disease when compared to the mice administered the formulation intraperitoneally that all died. Quantification of the cerebral parasite burden revealed a significant reduction of the parasite numbers in mice vaccinated intranasally compared to mice vaccinated intraperitoneally. Intranasal administration of the vaccine induced an immune response of a higher IL-10 to IL-12 ratio suggesting Th2-biased immune response that provide protection of the mice against the disease [97].

#### 4.7.2. Nanogels Application as Vaccine for Intranasal Administration for the Prevention of Mouth to Foot Disease

Mouth to foot disease is endemic in most countries of the world and a highly contagious viral infection of wild cloven-hoofed animals such as buffaloes, pigs, sheep, goats [99,100]. The disease causes huge economic loss in the affected countries and it is caused by picornavirus. The disease is characterized by a reduction in milk production, suppressed livestock growth, loss of appetite, redness of the oral mucosa, excessive salivation and high fever, [99,100]. The best approach for the protection of animals against mouth to foot infection is by the administration of vaccine via the nasal mucosa which is characterized by a highly vascularized area with large surface area. Çokçalışkan et al. developed gel formulation (sponge and gel) from different types and concentration followed by the incorporation of the whole inactivated foot to mouth disease virion (Table 1) [101]. The formulations induce significant systemic immune responses. Administration of the formulation intranasally, induce nasal IgA titers after second boosting in guinea pigs in vivo when compared to the administered antigen subcutaneously which did not induce any significant IgA response. The result suggested that the chitosan-based nasal formulations prevent the viral infection in upper respiratory mucosa by inducing IgA responses. The immune response was higher in the soluble chitosan when compared to the base chitosan formulations with lower pH. The finding revealed that at low pH, the foot to mouth disease virion dissociates into pentamers resulting in the reduced ability of the virus to induce antibody response. In vivo studies in cow further revealed that the formulation induced nasal IgA, serum IgG responses and neutralized antibody responses which are important for protection. No side effects were observed in all the formulations. The gels dilution with the mucus secreted in the nasal cavity of the cattle resulted in the enhanced resistance of the formulation to mucociliary clearance. The sponge formulation was retained longer in the mucosa resulting in an extended contact of the antigen and an increased cell uptake. The molecular weight of the chitosan did not have any significant effect. However, formulation with higher pH enhanced the immune response [101].

## 5. Conclusions

Intranasal administration of therapeutics using nanogels can bypass BBB resulting in good drug uptake in the brain with excellent neuroprotective effect. Nanogels have been reported to be useful in the enhanced delivery of insulin to the brain resulting in a significant amount of the insulin in the anterior and cerebellar regions of the mouse brain. The result revealed that nanogel is a potential carrier for the treatment of mild to moderate Alzheimer disease resulting in improved cognitive performances and brain function. Nanogels are also useful for administration of antipsychotic drugs with poor bioavailability via intranasal route for the treatment of schizophrenia. The nature of the polymer used to prepare the gel influenced the drug dissolution rate in release medium, its ability to adhere to mucosal tissues and its capacity to transiently open the tight junctions influenced the drug absorption of the drug from the nanoparticles on administration of the formulation intranasally. Nanogels are also potential carrier for the delivery of therapeutics with rapid nasal mucociliary clearance. Incorporation of anti-migraine drug, lidocaine hydrochloride that is characterized by rapid nasal mucociliary clearance resulted in an improved bioavailability of drug in vivo.

Nanogels have also been employed for the delivery of anti-depression drug resulting in a high brain/blood ratio of the drug after administration of the formulation intranasally. The olfactory and trigeminal nerve systems are involved in the nose to brain transportation of drugs to the olfactory bulb or trigeminal region in which the drug enter into other brain regions by diffusion thereby bypassing systematic circulation. Nanogels are effective as targeted carriers that protect vaccine antigens from degradation in vivo and also act as a vaccine delivery. The advantages of nanogels as vaccine delivery systems are their good loading capacity, stability and non-toxicity. The vaccine formulations of nanogels induced antibody responses which are useful for protection against viral, bacterial and lifestyle diseases. The formulation induced humoral and cellular immune response. The formulations were not transported to the brain revealing the safety of nanogels. Despite the great potentials of nanogels in drug delivery and in vaccine formulations, most of the reports are preclinical studies indicating that there is a need for these formulations to reach clinical studies. Major advances in the development of nanogel-based vaccines and drug delivery systems can be expected in the near future. However, a detailed evaluation of the current vaccines and a good understanding of disease pathology and biomaterials will provide targeted and effective delivery systems for preventive and therapeutic applications.

## Figures and Tables

**Table 1:** Nanogel gel for intranasal administration.

Application	Polymer	Drug/Vaccine	Biological Outcomes	References
Alzheimer disease	Poly(*N*-vinyl pyrrolidone)	Insulin	Protection of the loaded insulin from protease degradation. Enhanced uptake of insulin in the brain.	[44,48]
Alzheimer disease	Chitosan	Piperine	In vivo studies showed that the formulation enhanced the cognitive functions of the mice.	[52]
Schizophrenia	Chitosan	olanzapine	Good nasal absorption of the drug.	[55]
Migraine	Hydroxypropyl methyl cellulose	lidocaine hydrochloride	The drug targeting index of olfactory and ventricle after nasal gel was enhanced compared to the nasal spray.	[58]
Depression		Venlafaxine	The prolonged duration of action of the loaded drug.	[61]
Depression	Alginate	Venlafaxine	Increased permeation of drug and the mucosal absorption.	[62]
Depression	Chitosan	Selegiline	Reduced oxidative stress and restoration of the activity of the mitochondrial complex in vivo.	[64]
Hypertension	Chitosan	Amlodipine besylate	The formulation did not exhibit drug toxicity on the sheep nasal mucosa in vitro.	[66]
Cancer	Chitosan	Leuprolide acetate	Sustained drug release.	[67]
HIV	Chitosan	Didanosine	The drug concentration in the cerebrospinal fluid, olfactory bulb and brain in vivo was high.	[76]
*S. pneumoniae* infection	Pullulan	pneumococcal surface protein A antigen	formulation induced humoral and cellular immune responses.	[83,84,85]
Influenza	Pullulan	*Clostridium botulinum* type-A neurotoxin	The vaccine did not accumulate in the brain.	[88]
Influenza	Pulllulan	tumor necrosis factor-α	The formulation induced systemic IgG1 and mucosal IgA antibodies. It was effective in protecting the mice against a lethal challenge of A/PR/8/34 (H1N1) influenza virus.	[89]
Obesity	Pullulan	Ghrelin-pneumococcal surface protein A	The vaccine did not alter food intake in immunized mice. Peroxisome proliferator-activated receptor gamma expression in adipocytes was increased in the mice immunized with the formulation.	[93]
Veterinary application (Encephalomyelitis)	Chitosan and alginate	recombinant NcPDI.	The formulation protected the mice against the disease.	[97]
Mouth to foot disease	Chitosan	inactivated foot to mouth disease virion	The formulation prevented the viral infection in upper respiratory mucosa by inducing IgA responses.	[101]
